# Supplementary material for: Favorable Biological Performance Regarding the Interaction between Gold Nanoparticles and Mesenchymal Stem Cells
Source: Int J Mol Sci. 2022 Dec 20;24(1):5. doi: 10.3390/ijms24010005 (PMC9819939; doi:10.3390/ijms24010005)
Supplement: Supplementary file 1 [file ijms-24-00005-s001.zip › ijms-2071382-supplementary.pdf]

Favorable Biological Performance Regarding the Interaction Between Gold Nanoparticles and Mesenchymal Stem Cells

Supplementary data

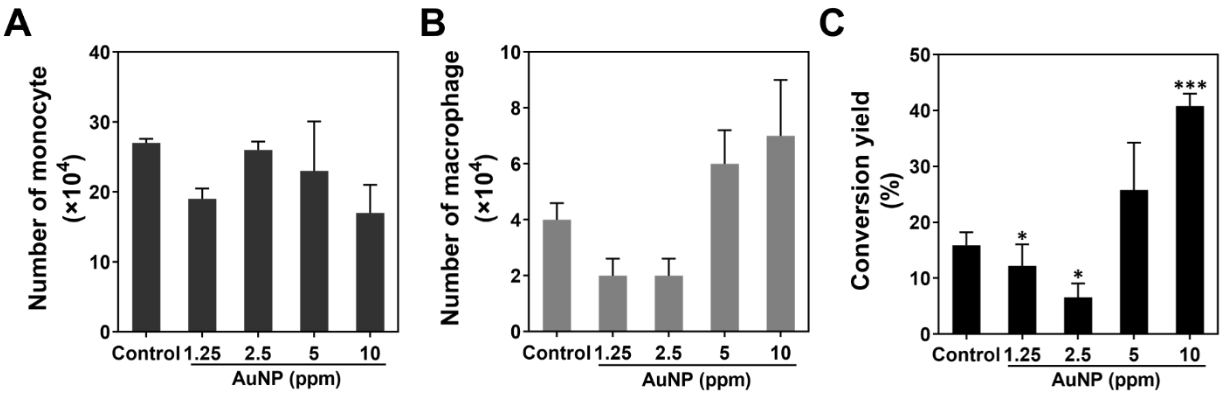

Figure S1. The ratio of monocyte conversion after 96 hours of AuNP treatments. (A) The monocyte number and (B) the macrophage number in each treatment is displayed. (C) The ratio of monocytes converted to macrophages was quantified, and the results explain that the conversion ratio in the AuNP 1.25 and 2.5 ppm groups was significantly lower than the other treatments. The above results are demonstrated as mean ± SD (n = 3). * $p < 0.05$, *** $p < 0.001$: compared to the Control group (TCPS).

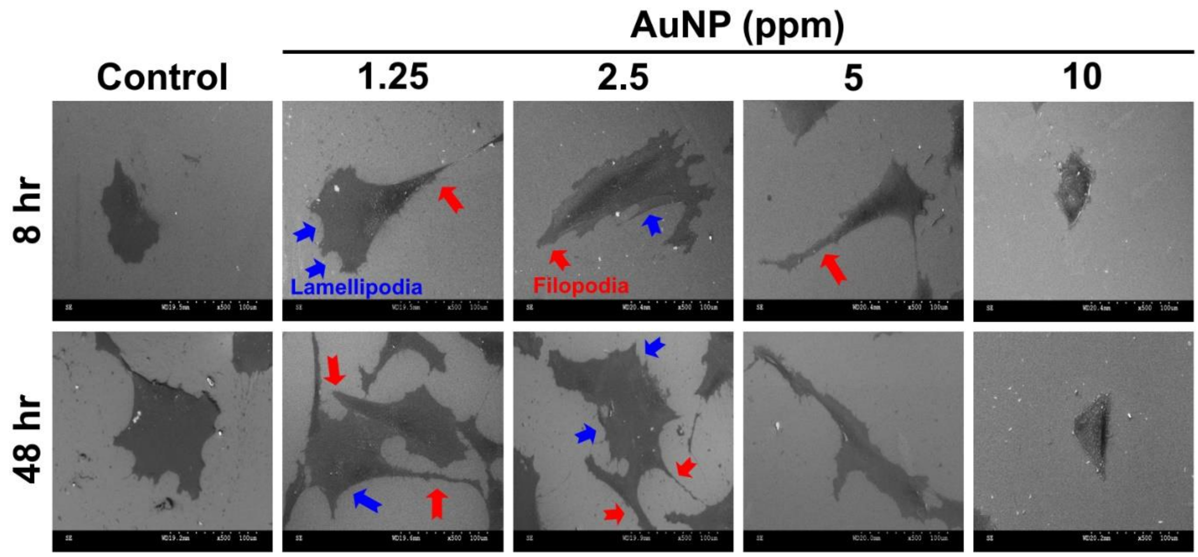

Figure S2. Cell morphology of MSCs at 8 and 48 hours was observed through SEM. The SEM images of MSCs incubated with various concentration of AuNP solution are displayed. The red arrows: filopodia. The blue arrows: lamellipodia. The morphology of filopodia and lamellipodia can be clearly observed in both the AuNP 1.25 and 2.5 ppm groups. Scale bar = 100 μm. The images were selected based on one of three independent experiments.

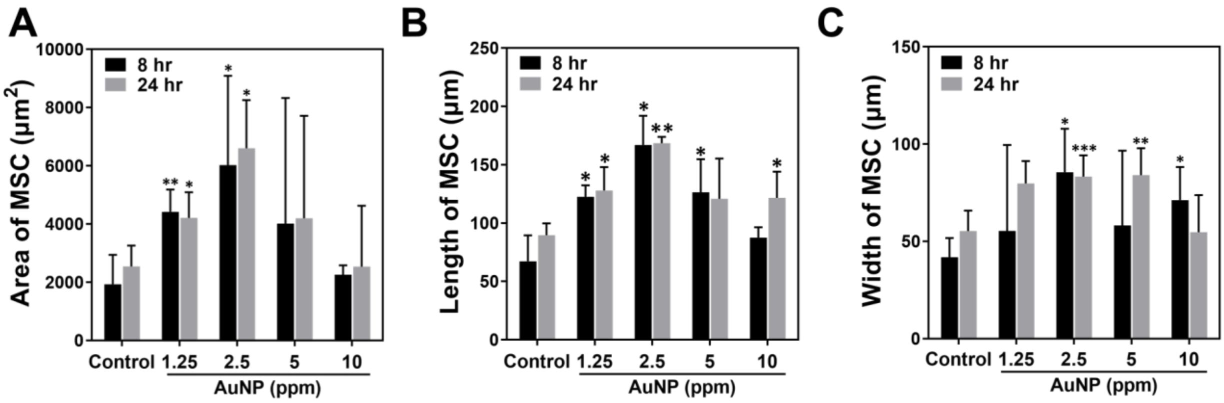

Figure S3. (A) The area of MSCs was analyzed, while (B) the length and (C) width of MSCs were also investigated. The above results are demonstrated as mean ± SD (n = 3). * $p < 0.05$, *** $p < 0.001$: compared to the Control group (TCPS).

Cell viability (OD 570_{nm})	24 hr	48 hr	72 hr
Control	0.35	0.58	0.92
AuNP 1.25 ppm	0.37 *	0.62 ***	1.04 ***
AuNP 2.5 ppm	0.37*	0.62 ***	1.07 ***
AuNP 5 ppm	0.25 ***	0.54 ***	0.65 ***
AuNP 10 ppm	0.15 ***	0.17 ***	0.17 ***

Table S1. The OD value of cell viability within MSCs at 24, 48 and 72 hours. * $p < 0.05$, *** $p < 0.001$: compared with the control (TCPS).
